# Frequency and outcome of treatment in polycystic ovaries related infertility

**DOI:** 10.12669/pjms.313.8003

**Published:** 2015

**Authors:** Farzana Arain, Nesreen Arif, Hafeez Halepota

**Affiliations:** 1 Dr. Farzana Arain, Associate Professor, Taif Medical College, Taif, Saudi Arabia; 2 Dr. Nesreen Arif, Assistant Professor, Taif Medical College, Taif, Saudi Arabia; 3 Dr. Hafeez Halepota, Professor of Gynae & Obstetric Department, Muhammad Medical College Hospital, Mirpurkhas, Sindh, Pakistan

**Keywords:** Polycystic Ovaries, Infertility, Ovulation induction, Laparoscopic ovarian drilling

## Abstract

**Background:**

Infertility is defined as inability of couple to conceive after one year of unprotected intercourse. The prevalence of infertility in Pakistan is 21.9%. The most common cause of medically treatable infertility is polycystic ovarian syndrome (PCO). This study was conducted to see the frequency and outcome of treatment in PCOs related infertility in infertile couples coming to Mohammad Medical College Hospital, Mirpurkhas, Sindh.

**Methods:**

This prospective observational study was conducted at Muhammad Medical College for three years from 2005 to 2008. Total 1289 infertile couples were included in this study.

**Result:**

The frequency of PCOs in female related infertility was 38.5%. Other causes of female infertility were in the frequency of 44% pelvic inflammatory disease, 12.3% endometriosis, 2.9% hyperprolactenemia, and 1.35% hypothyroidism. Patients with PCOS were given different treatment modalities. One hundred fifty patients with PCO were given ovulation induction with clomephene citrate and out of them 109 (72%) conceived. Sixty three women were given combination of clomephene citrate and Metformin. Out of them 50 (79%) conceived. Five patients were given gonadotrophins, Out of them 2 (40%) patients conceived. Five patients had laparoscopic drilling out of them 3 (60%) conceived.

**Conclusion:**

In contrast to the literature review Polycystic Ovarian Syndrome turned out to be the second most common cause of female related infertility. But as the international literature shows it had very good out come after medical and /or surgical treatment.

## INTRODUCTION

Infertility is a worldwide problem which has profound social and emotional implications for the individual concerned. WHO-DHS Comparative Report in 2004 states that more than 186 million ever-married women in developing countries (excluding China) were infertile because of primary or secondary infertility, Infertility, and Childlessness in Developing Countries as per DHS Comparative Reports.^1^

Although Pakistan is among the currently most populous countries of the world, and has a population growth rate of around 2%, it also has high rate of infertility (21.9%); 3.5% primary and 18.4% secondary the prevalence of infertility in Pakistan is 21.9%.^2^

Infertility in women has many possible causes. The most common cause of treatable infertility is polycystic ovarian syndrome, common in young women and cause of an ovulatory infertility in 70% cases.^3^ The World Health Organization classification offers a useful frame for diagnosis and treatment. Polycystic ovary syndrome is the most common cause of oligo ovulation and anovulation.^4^ Poly cystic ovarian syndrome (PCOS) is a common endocrine disorder which causes anovulatory infertility.^5^ In PCOS Increased ovarian androgen production leads to premature adrenarche, menstrual irregularity, acne, hirsutism, and infertility by means of elevated luteinizing hormone to follicle stimulating hormone production and hyperinsulinemia. 6

Polycystic ovarian syndrome is a heterogeneous condition proposed diagnostic criteria for poly cystic ovaries is as follows, first criteria is –menstrual irregularity due to oligo or an ovulation, clinical and/or biochemical signs of hyperandrogenesim. Exclusion of other disorder e.g. non classic congenital renal hyperplasia & androgen secreting tumors.^7^ For Rotterdam criteria to diagnose PCO two out of three following condition are required to present. Oligo-or an ovulation, clinical and/or biochemical signs of hyperandrogenesim and prescence of poly cystic ovaries on ultrasound.^8^

According to Androgen Excess Society (AES) the diagnostic criteria to diagnose PCO all the following should be present i.e. 1, Clinical and/or biochemical signs of hyperandrogenism. 2, Ovarian dysfunction – oligo-an ovulation and/or polycystic ovaries on ultrasound 3, Exclusion of other androgen excess or ovulatory disorders.^9^

Couples who have been trying to conceive for 1-2 years should be investigated. Detailed history, Examination and investigation of both partners should be taken. In females, history of normal menstrual cycle is suggestive of ovulation. Confirmation of ovulation is obtained by mid luteal Serum progesterone level (>30 nmol/LAndrostenedione level, FSH and LH levels, GnRH stimulation. Exclude all other disorders that can result in menstrual irregularity and hyperandrogenism, including adrenal or ovarian tumors, thyroid dysfunction, congenital adrenal hyperplasia, hyperprolactinemia, acromegaly, and Cushing syndrome.^10^

Baseline screening laboratory studies for women suspected of having PCOS include Thyroid function test (e.g., TSH, free thyroxin), Serum prolactin level, Total and free testosterone levels, Free androgen index, Serum HCG level, Cosyntropin stimulation test serum 17-hydroxyprogesterone (17-OHPG) level, Urinary free cortisol (UFC) and creatinine levels, Low-dose dexamethasone suppression test, Serum insulin like growth factor (IGF)–1 level Other tests used in the evaluation of PCOS include the following on testing, Glucose level and Insulin level.^11^ Regarding management life style modifications are considered first-line treatment for women with PCOS. Such changes include the following;^12^ Diet, Exercise, Weight loss. In obese women with PCOs a loss of 5-10% of body weight may be enough to restore reproductive function in 55-100%women within 6 month.^13^

Clomiphene citrate is an orally active synthetic compound with estrogenic as well as anti estrogenic properties, which has traditionally been used as the treatment of choice in women with an ovulation in Poly cystic ovary syndrome. Ovulation is expected to occur in 80% and pregnancy in (35-40% women on clomiphene.^14^ Approximately 20-25% of women show no response to clomiphene Citrate and are considered to be resistant.

The strong association between an ovulation and insulin resistance has led to speculation that lowering insulin level would lead to improvement in the clinical and metabolic profile of woman with PCOs. Metformin is more effective in achieving ovulation in women with PCOs in comparison with placebo.^15^

Laparoscopic ovarian drilling (LOD) by diathermy or laser is another treatment option for women with an ovulation associated with PCOs, unipolar coagulation current is used to deliver four puncture to a depth of 4 mm in each ovary.^4^ A Cochrance review showed that ongoing cumulative pregnancy rate following LOD was similar to those obtained with 3-6 cycles of gonadotrophins.^4^

Laparoscopy ovarian drilling has obvious comparative advantage to competitive chemotherapeutic agents. Reduction in overall cost of treatment and multiple gestations implies that it may be the treatment of choice in women with CC-resistant PCOS.^16^

### Gonadotrophins

Treatment with gondotroprophins is contemplated when woman either do not respond to clomiphene or fail to conceive after 6-12 ovulatory cycle. In women with PCOs treatment with gandotrophins results in cumulative pregnancy rate of 40-50%.^17^

### GNRH analogues

GNRH analogues have been used in conjunction with gandotrophins to achieve pituitary down regulation and to facilitate cycle control. Systemic review of three randomized controlled trails (RCTs) failed to reveal any added benefit associated with the use of GNRH analogs with gonadotrophins in terms of pregnancy rates.^18^

## METHODS

This prospective observational study was conducted at Mohammad Medical College hospital for the period of three years from January 2005 to December 2008. Twelve hundred eighty nine couples were included in this study. All the infertile women was assessed by Detailed history, Full examination, Serum day-21 progesterone levels, Ultrasound of pelvis, Tubal patency test either by laparoscopy or hysterosalpingography and different hormonal analysis was done. This included Thyroid profile Serum prolactin levels and others. Serum FSH, Day–21 progesterone, serum testosterone level and other like GnRH stimulation testing, Glucose level and Insulin level.

Age, height, weight, BMI, was taken as female variable. All investigations results were evaluated. We used Rotterdam criteria to diagnose PCOS. Different treatment was given according to the investigation result, which includes the following.

### 1. Ovulation induction with clomiphene citrate

Tablet clomephene citrate 5o mg daily for five days, from 2^nd^ day of menstrual cycle for five days when no result found then the dose increased to 100 mg. in second cycle, from second day of menstrual cycle for five days. When no result was found the dose of clomiphene citrate was increased to 150 mg daily from 2^nd^ day of menstrual cycle for five days in third menstrual cycle. When the result was still not found, then for next three cycle dose was maintained 150 mg daily for five days.

### 2. Metformine with clomiphene citrate

Metformine with clomiphene citrate was used in those women who was obese; dose of Metformin was 250 mg twice a day daily with clomiphene citrate in same dosage as above for six month duration.

### 3. Gonadtrophins for ovulation induction

When there was no induction with Clomiphene citrate, injectable gonadotrophin was given for induction of ovulation by following regimen injectable gonadotrophins were given 75 IU by intramuscular injection. The injection started from the second day of menstrual cycle daily for 10 days. Ultrasound monitoring was done to exclude ovarian hyperstimulation monitoring was done twice a week. The injection was continued till on ultrasound one or two mature follicles were seen about 18 mm. At that point an injection of HCG 5000 IU was given to induce ovulation approximately 36 hours later. This regimen was tried for three to six months, when no result found couple were referred for in vitro fertilization.

### 4. Ovarian drilling with clomiphen citrate

When a course of clomiphene citrate fails to result in conception, human menopausal gonadotrophins or laparoscopic ovarian drilling was offered to the couple as a second line treatment. Couples went for ovarian drilling who did not afford gonadotrophines injections plus not only affordability they could not come for monitoring from far areas of Thar.

We did laparoscopic ovarian drilling by using monopolar diathermy. The operation was carried via three areas of entry. A 10 mm laparoscope was inserted via primary sub – umbilical trocar, with two additional 5 mm ports, a grasping forceps were used to hold the ovarian ligament for manipulation of the ovaries. Diathermy needle was introduced via the other port. A laparoscopic ovarian diathermy needle, of 8 mm in length and 2 mm in diameter was used. A standardized monopolar coagulation current at approximately 30 waltz were used. Two to four holes were made in each ovary. The craters created were 3 to 4 mm in diameter and 8 mm in depth. At the end of procedure the operated site was irrigated with Hartman’s solution.

After two months of ovarian drilling, their D- 21 progesterone levels were checked if not indicative of ovulation then clomiphene citrate was used to induce ovulation. Using the same protocol as mentioned earlier.

Success rate we defined a successful outcome as one in which pregnancy occurred within 12 months for two reasons. First, women want to know how long it might take for conception to occur, so a period of 12 months seems reasonable, beyond which she may be advised to pursue alternative treatment such as gonadotrophins ovarian stimulation or in vitro fertilization. Out come in terms of cumulative singleton live birth rate was assessed.

### Exclusion criteria

All infertile couples who had other causes of infertility than PCOS were excluded from the study.

## RESULTS

Out of 5667 women attending gynecology OPD during study period, 1289 (22.7). Couples presented with infertility. Female factors were recognizable in 446 (34.6). Among them pelvic inflammatory disease which refers exclusively to infection of female upper genital tract (the female structures above the cervix) was seen in 200 (44%) women, which seems to be the leading cause in this study. Second cause of female infertility was PCO which was seen in 172 (38.5%) of women. Other causes of female infertility were in the frequency of 12.3% endometriosis, 2.9% hyperprolactenemia i.e., an excessive amount of prolactin in the blood, usually caused by a pituitary adenoma but sometimes caused by endocrine side effects related to certain antipsychotic medications. In women it is usually associated with galactorrhea and secondary amenorrhea; and 1.35% hypothyroidsm. ie: Deficiency of thyroid hormone which is normally made by the thyroid gland which is located in the front of the neck.

All patients other than PCO were excluded from the study and further outcome of treatment was observed in PCO patient. (Table-I)

**Table-I:** Causes of female factor infertility.

	Total No. 446	
Pelvic Inflammatory disease	200	44%
Polycystic Ovarian Syndrome	172	38.5%
Endometriosis	50	11.2%
Hyperprolactinemia	9	64%
Hypothyroidism	5	35%
Unexplained	10	2.24%

These patients were given different treatment modalities. One hundred fifty patients with PCO were given ovulation induction with clomephene citrate and out of them 109 (72%) conceived. Sixty three women were given combination of clomephene citrate and Metformin. Out of them 38(60%) conceived. Five patients have gonadotrophins. Out of them 1 (20%) patients conceived. Five patients had laparoscopic drilling out of them 3 (60%) conceived. (Table-II).

**Table-II:** Different methods of treatment and no. of patient conceived.

Method of Treatment Given	No. of patients treated	No. of patients conceived	Percentage
Ovulation Induction with clomephene citrate	150	109	72%
Metformin+ Clomiphene Citrate	63	38	79%
Gonadotrophins	5	1	20%
Laparoscopic ovarian drilling	5	3	60%

## DISCUSSION

In this study PCOs related infertility turned out to be the second most common cause of female factor related infertility that is 38.5%. A study was done to see the prevalence of PCOS in Pakistan it turned out to be 20.7%. This retrospective study was carried out at Women Clinic and fertility advisory center, Islamabad from Jan. 1997 to May 1999 on 52 women having polycystic ovary syndrome with fertility deprivation. The diagnosis and management of such cases has been analyzed. The prevalence of PCOS in this study was found 20.7%.^19^

Clomiphene Citrate was given as the first line of treatment. With this drug the cumulating pregnancy rate approaches to that of normal woman as in on clomiphene, the pregnancy rate was of about 72%. Almost same results has been seen in a study where it was concluded that Women under 35 with PCOS should have about a 10-15% chance for pregnancy per month with Clomid treatment (for about 3-4 months) - if they are ovulating with the Clomid (sperm and fallopian tubes must be normal for these statistics.^20^ The patient who was resistant to Clomiphene citrate alone. They have given combination of Clomiphene citrate and Metformin, and it showed more increased pregnancy rate. As it was also seen in recent meta analysis of eight randomized controlled trials has shown that Metformin plus Clomiphene citrate may be superior to Clomiphene alone.^21^ In UK the National Institute of Clinical excellence (NICE 224)) guide line support the use of Metformin in association with Clomiphene in an ovulatomy infertility. In another study combination of clomephene citrate and Metformin showed increased pregnancy rate than clomiphene citrate alone.^12^ Another study showing and recommending that Metformin combined with clomiphene citrate may increase ovulation rates and pregnancy rates but does not significantly improve the live birth rate over that of clomiphene citrate alone. Metformin may be added to clomiphene citrate in women with clomiphene resistance who are older and who have visceral obesity.^22^

Laparoscopic drilling (LOD) showed promising result, about 60% patient conceived during this study period after LOD. Same result showed by another study, which was carried out to determine the effectiveness and safety of minilaparotomy and ovarian drilling for sub fertile women with clomiphene resistant polycystic ovarian syndrome. Fourteen out of 16 patients ovulated within 6 months of ovarian drilling. Eleven patients conceived during 6 months follow up, with pregnancy rate of about 68.75%. The greatest success rate being in women with shorter length of infertility, patients who were young and had secondary infertility so it was concluded that Ovarian drilling is a powerful tool in the treatment of polycystic ovaries.^20^ A randomized study suggested that combined Metformin/letrozole and bilateral ovarian drilling are similarly effective as second-line treatment in infertile women with clomiphene citrate–resistant PCOS. In this study, 146 patients were given Metformin and letrozole, and 73 underwent bilateral ovarian drilling. There was significant reduction in testosterone, fasting insulin, and ratio of fasting glucose to fasting insulin in the Metformin/letrozole group. There was significant reduction in follicle-stimulating hormone (FSH), luteinizing hormone (LH), and ratio of LH to FSH in the bilateral drilling group. There was no significant difference between the patients in the 2 groups regarding cycle regularity, ovulation, pregnancy rate, and abortion rate.^23^

Gonadotrophins was used as another treatment modality, with very much hormonal and ultrasound monitoring to avoid ovarian hyperstimulation syndrome. Conception rate was less in comparison to other treatment modalities specially LOD. But in contrast to this, a study in 2010, states that one has to consider that LOD might lead to a decrease in the ovarian reserve due to surgical destruction of the ovarian capsule/to adhesion formation both possibly resulting in impairment of infertility. Thus LOD must remain second or third line treatment option.^24^ In another study it has been seen that in clomephene citrate resistant cases, insulin lowering agents are safer than laparoscopic ovarian drilling. It has been concluded that: To avoid the risk of adverse effects of LOD preference may be given to the use of rosiglitazone and CC therapy in patients of PCOS resistant to CC.^25^ Another study, a double-blind trial by Legro et al., found that letrozole is more effective than clomiphene in the treatment of infertility in PCOS. Based on treatment periods of up to five cycles, the study, which involved 750 anovulatory women with PCOS, found that the birth rates for letrozole and clomiphene were 27.5% and 19.1%, respectively. The rate of congenital abnormalities and the risk of pregnancy loss in the letrozole and clomiphene groups were found to be comparable, although the likelihood of twin births was lower with letrozole.^26^

In contrast to this another study compares the hormonal-metabolic profile and reproductive outcome in PCO patient, who were comparing the case of clomiphene resistant by women receiving Metformin and those undergoing laparoscopic ovarian drilling. The authors concluded that although Metformin results in a better attenuation of insulin resistance, laparoscopic ovarian drilling is associated with higher rates of ovulation and pregnancy.^27^

## CONCLUSION

With appropriate treatment in properly selected cases PCOS is really a medically treatable cause of female’s related infertility. Weight loss, exercise, and lifestyle modifications have been proven effective in restoring ovulatory cycles and achieving pregnancy in overweight women with polycystic ovary syndrome (PCOS) and should be the first-line option for these women. Clomiphene Citrate is the treatment of choice as 1st line therapy in PCOs related infertility. Clomiphene Citrate and Metformine turned out to be more effective than Clomiphene Citrate alone in clomiphene resistant cases. Laparoscopic drilling is better option than Gonadotophins. When extensive monitorining is not acceptable by the couples due to financial problems or due to residence in remote areas, but still more work is required in this regards as expertise for laparoscopic ovarian drilliling are also not freely available everywhere.

## References

[1] Rutstein SO, Shah IH (2004). Infecundity, Infertility, and Childlessness in Developing Countries. DHS Comparative Reports No 9.

[2] Shaheen R, Subhan F, Sultan S, Subhan K, Tahir F (2010). Prevalence of Infertility in a Cross Section of Pakistani Population. Pak J Zool.

[3] Adma J, Pson DW, Frank (1986). Prevalence of poly cystic ovaries in women with an Ovulation and idio pathic hirsutism. Br Med J.

[4] Haq F, Rizvi J (2008). Infertility and polycystic ovarian syndrome-a study of association b/w body mass index and intra family miscarriage. Gynecol Obstet Invest.

[5] Kousta E, White DM, Cela E, McCarthy MI, Franks S (1999). The prevalence of polycystic ovaries in women with infertility. Hum Reprod.

[6] Sam S, Dunaif A (2003). Polycystic ovary syndrome: syndrome XX?. Trends Endocrinol Metab.

[7] Zawadski JK, Dunaif A, Dunaif A, Givens JR, Haseltine FP, Merriam GE (1992). Diagnostic criteria for polycystic ovary syndrome: Towards a rational approach. Polycystic Ovary Syndrome (Current Issues in Endocrinology and Metabolism).

[8] (2004). Rotterdam ESHRE/ASRM-Sponsored PCOS consensus workshop group. Revised 2003 consensus on diagnostic criteria and long-term health risks related to polycystic ovary syndrome (PCOS). Hum Reprod.

[9] Azziz R, Carmina E, Dewailly D, Diamanti-Kandarakis E, Escobar-Morreale HF, Futterweit W (2009). The Androgen Excess and PCOS Society criteria for the polycystic ovary syndrome: the complete task force report. Fertil Steril.

[10] Goodarzi MO, Shah NA, Antoine HJ, Pall M, Guo X, Azziz R (2006). Variants in the 5alpha-reductase type 1 and type 2 genes are associated with polycystic ovary syndrome and the severity of hirsutism in affected women. J Clin Endocrinol Metab.

[11] Vause TD, Cheung AP, Sierra S, Claman P, Graham J, Guillemin JA (2010). Ovulation induction in polycystic ovary syndrome. J Obstet Gynaecol Can.

[12] (2008). Consensus on infertility treatment related to polycystic ovary syndrome. Fertil Steril.

[13] Farquhar C, Vandekerckhove P, Lilford R (2001). Laparoscopic “drilling” by diathermy or laser for ovulation induction in anovulatory polycystic ovary syndrome. Ostetrics & Gynaecology, National Women's Hospital, Claude Rd, Epsom. Cochrane Database Sys Rev.

[14] (2009). Spremovic-Radenovic Svetlana. Medical treatment of infertility. Acta Clinica.

[15] Nazir F, Saeed S, Malik M, Aziz H, Aliya S, Rana S (1999). Polycystic ovary syndrome (PCOS) - Diagnosis and Management in Fertility Deprivation. Pak J Obstet Gynaecol.

[16] Oriji VK (2010). Laparoscopic ovarian drilling verses medical treatment in management of clomephene citrate poly cystic ovarian syndrome. World J Laparoscopic Surg.

[17] Clark AM, ledger, Galletly C, Tomlinson L, Blaney F, Wang X (1995). Weight Loss result in significant improvement in pregnancy and ovulation rates in anovulatory obese women. Hum Reprod.

[18] Zain MM, Jamaluddin R, Ibraheem A, Nomn RJ (2009). Comparison of clomephene citrate, metformaine or the combination of both for the first-line ovulation induction, achievement of pregnancy, and live birth in Asian women with polycystic ovary syndrome. Fertil Steril.

[19] Palomba S, Orio F, Falbo A, Russo T, Tolino A, Zullo F (2007). Clomiphene Citrate Versus Metformin as First-Line Approach for the Treatment of An ovulation in Infertile Patients with Polycystic Ovary Syndrome. J Clin Endocrinol Metab.

[20] Abbasi AN, Sultana A, Izhar R, Begum S, Razaq S (2003). Minilaparotomy and Ovarian Diathermy Drilling for Clomiphene resistant Poly cystic Ovarian disease. J Ayub Med Coll Abottabad.

[21] Hamed HO, Hasan AF, Ahmed OG, Ahmed MA (2010). Metformin versus laparoscopic ovarian drilling in clomiphene- and insulin-resistant women with polycystic ovary syndrome. Int J Gynaecol Obstet.

[22] Vause TD, Cheung AP, Sierra S, Claman P, Graham J, Guillemin JA (2010). Ovulation induction in polycystic ovary syndrome. J Obstet Gynaecol Can.

[23] Abd Elgafor I (2013). Efficacy of combined Metformin-letrozole in comparison with bilateral ovarian drilling in clomiphene-resistant infertile women with polycystic ovarian syndrome. Arch Gynecol Obstet.

[24] Ott J, Kurz C, Nouri K, Wirth S, Vytiska-Binstorf E (2010). Pregnancy outcome in women with polycystic ovary syndrome comparing the effects of laparoscopic ovarian drilling and clomiphene citrate stimulation in women pre-treated with metformin. Reprod Biol Endocrinol.

[25] Roy KK, Baruah J, Sharma A, Kumar S (2010). A prospective randomized trial comparing the clinical and endocrinological outcome with rosiglitazone versus laparoscopic ovarian drilling in patients with polycystic ovarian disease resistant to ovulation induction with clomiphene citrate. Arch Gynecol Obstet.

[26] Emery G (2014). Letrozole Produces More Babies in Women with Polycystic Ovary Syndrome: Study. Medscape [serial online].

[27] Legro RS, Brzyski RG, Diamond MP, Coutifaris C, Schlaff WD, Casson P (2014). Letrozole versus clomiphene for infertility in the polycystic ovary syndrome. N Engl J Med.

